# Negative emotion provides cues for orienting auditory spatial attention

**DOI:** 10.3389/fpsyg.2015.00618

**Published:** 2015-05-12

**Authors:** Erkin Asutay, Daniel Västfjäll

**Affiliations:** ^1^Division of Applied Acoustics, Department of Civil and Environmental Engineering, Chalmers University of Technology, Gothenburg, Sweden; ^2^Department of Behavioral Sciences and Learning, Linköping University, Linköping, Sweden; ^3^Decision Research, Eugene, OR, USA

**Keywords:** auditory perception, emotion, auditory spatial attention, covert spatial orienting, dot-probe task

## Abstract

The auditory stimuli provide information about the objects and events around us. They can also carry biologically significant emotional information (such as unseen dangers and conspecific vocalizations), which provides cues for allocation of attention and mental resources. Here, we investigated whether task-irrelevant auditory emotional information can provide cues for orientation of auditory spatial attention. We employed a covert spatial orienting task: the dot-probe task. In each trial, two task-irrelevant auditory cues were simultaneously presented at two separate locations (left–right or front–back). Environmental sounds were selected to form emotional vs. neutral, emotional vs. emotional, and neutral vs. neutral cue pairs. The participants’ task was to detect the location of an acoustic target that was presented immediately after the task-irrelevant auditory cues. The target was presented at the same location as one of the auditory cues. The results indicated that participants were significantly faster to locate the target when it replaced the negative cue compared to when it replaced the neutral cue. The positive cues did not produce a clear attentional bias. Further, same valence pairs (emotional–emotional or neutral–neutral) did not modulate reaction times due to a lack of spatial attention capture by one cue in the pair. Taken together, the results indicate that negative affect can provide cues for the orientation of spatial attention in the auditory domain.

## Introduction

The human brain is equipped with various mechanisms like executive functions and selective attention to deal with the vast amount of information flow from the external world in a seemingly effortless manner (Driver, 2001). These mechanisms grant the brain to prioritize a subsection of the total external stimulation depending on its perceptual salience (bottom-up effects) and momentary relevance to the organism (top-down effects). A growing body of empirical evidence suggests that emotional significance of objects and events provide cues for the allocation of attention and mental resources (Vuilleumier, 2005). In other words, emotional stimuli, by forming a special case of high-salience, may capture attention easier than non-emotional stimuli (Phelps and LeDoux, 2005; Vuilleumier and Driver, 2007). Emotional stimuli with their perceptual properties and biological significance can lead to attentional prioritization. Here, we investigated whether the auditory spatial attention could be modulated by the emotional significance of sounds.

It has been argued that emotionally significant stimuli influence attention and enhance sensory processing via fast neural routes to sensory cortices (Phelps, 2006; LeDoux, 2012). However, much of the evidence on the affective modulation of perception and attention is in the visual domain (Vuilleumier and Driver, 2007). Behavioral studies in the visual modality have provided evidence that emotional information may capture attention using various experimental paradigms such as attentional blink, visual search, and cueing (for a review, see Yiend, 2010). The role of emotional processing in auditory perception and attention, however, is a much less studied area. Nevertheless, a growing body of evidence indicates that emotion can influence auditory processing. The activation in the auditory cortex is enhanced in response to complex emotional auditory stimuli (Plichta et al., 2011). Negative emotion induced by visual stimuli can affect auditory event-related-potentials (ERPs) as early as 20 ms (Wang et al., 2009). It was also found that learned emotional meaning of the auditory stimuli influences the early auditory processing and engages the auditory attention networks (Bröckelmann et al., 2011); and that the auditory spatial attention may be influenced by the emotional information evidenced by early ERPs around 100 ms (Pauli and Röder, 2008). Further, recent behavioral evidence has shown that loudness perception can be affected by emotional salience of auditory stimuli, which is acquired through low-level affective learning (Asutay and Västfjäll, 2012); and that change-detection performance in complex auditory environments is influenced by emotional significance of individual sounds (Asutay and Västfjäll, 2014).

In the present study, we investigated whether the emotional salience of task-irrelevant auditory stimuli can be used as an exogenous cue in a covert spatial orientation task; i.e., the dot-probe task (Posner, 1980; Yiend, 2010). The typical use of the dot-probe task in the visual modality requires a brief simultaneous presentation of one emotional and one neutral picture (i.e., cues) at opposite sides of a fixation point. The task is to detect, as quickly and accurately as possible, a neutral target (e.g., a dot) that follows the cue presentation. The target is presented in the previous location of either the emotional or the neutral cue. The relevant measure in the dot-probe task is the time it takes to locate the target. Results typically show faster responses to targets replacing emotional rather than neutral cues (Pourtois et al., 2004; Lipp and Derakshan, 2005). Here, we adapted the dot-probe task for the auditory domain. The participants were instructed to locate an auditory target emanating from one of two possible locations, which was preceded by the simultaneous presentation of two task-irrelevant auditory cues at separate locations. To the authors’ knowledge there is only one other study that employed an adapted version of the dot-probe task in the auditory domain (Bertels et al., 2010), which used spoken words as cues and found attentional bias for the negative words only when they were presented on the participants’ right side. Hence, Bertels et al. (2010) suggested that the dominant left hemisphere processing of linguistic information could account for this effect. Unlike the above-mentioned study, we used non-linguistic emotional auditory stimuli as cues. As was discussed above, previous research has shown that emotional information can capture attention in tasks like attentional blink (Anderson, 2005) and dot-probe (Pourtois et al., 2004). The main argument here is that emotional stimuli draw attention faster and in a more robust manner compared to neutral stimuli. This effect was mostly reported for negative stimuli like angry or fearful faces that points to an adaptive behavior for potential threats. Even though similar effects were reported for positive stimuli in some attentional tasks (e.g. Brosch et al., 2008), the findings seem to be more variable. Further, it has been suggested that positive stimuli broadens the attention and facilitates exploration of new information (Rowe et al., 2007).

In the current experiment, ecological sounds were used to form neutral-emotional (positive or negative), emotional–emotional and neutral–neutral auditory cue pairs presented at separate locations. We hypothesized that task-irrelevant negative stimuli when paired with neutral sounds would provide a cue to orient spatial attention; and it would manifest itself in faster localization of the target when presented at the same location as the negative cue compared to the neutral cue. Further, we expected that emotional–emotional and neutral–neutral pairs (i.e., same valence cues) would not cause an attentional bias due to the smaller difference in emotional saliency between them.

## Materials and Methods

### Participants

Twenty-three normal-hearing individuals (12 women, 11 men, mean age: 26.9 years, SD: 4.6) participated in the study. They gave their informed consent prior to the inclusion in the experiment and were compensated after the study. The experiment was conducted in accordance with the ethical standards in the Declaration of Helsinki, and was approved by the Västra Götaland regional ethics committee. The experiment was carried out in a dark, sound-attenuated room, where participants completed all materials individually. Sample size was approximately determined based on the results of the previous experiments on auditory-induced emotion carried out in our laboratory (e.g., Tajadura-Jiménez et al., 2010; Asutay and Västfjäll, 2012, 2014).

### The Auditory Stimuli

Six sounds were used in the experiment as task-irrelevant auditory cues. They (see Table 1) were selected to form three affective categories (positive, negative, and neutral). In order to introduce a certain degree of physical variance within the affective categories, we used two sounds for each category. Four of the sounds (woman screaming, woman yawning, fire alarm, and beverage bottle opening) were time-edited versions of the original IADS sounds (Bradley and Lang, 2007); and the other two were originally from freesound.org. One of them was a functioning microwave oven, while the other one was a laughing baby sound. All the auditory stimuli were time-edited to make sure that they have the same onset and offset times. They were 3-s long and sampled at 44.1 kHz. We also performed loudness equalization according to the fifth-percentile Zwicker loudness values (N5), which is suggested as a loudness index for temporally-varying, non-impulsive sounds (Fastl and Zwicker, 2007).

**TABLE 1:** **Emotional reactions and stimuli information**.

**Stimulus**	**Valence (95CI)**	**Arousal (95CI)**	**Mean Pitch (Hz)**	Mean pitch—start (Hz)*	Mean pitch—end (Hz)*	Max loudness level—start (phon)*	Max loudness level—end (phon)*
*Negative*							
Woman screaming	–0.81 (0.08)	0.66 (0.11)	1310	1743	928	79.4	77.6
Fire alarm	–0.54 (0.12)	0.48 (0.12)	437	606	431	76.8	78.7
*Neutral*							
Woman yawning	–0.05 (0.16)	–0.25 (0.17)	325	378	257	77.9	78.8
Microwave oven	–0.09 (0.13)	–0.14 (0.12)	350	973	60	77.1	77.5
*Positive*							
Baby laughing	0.57 (0.14)	0.32 (0.12)	622	307	1084	78.7	76.9
Beverage bottle opening	0.33 (0.11)	0.08 (0.14)	2306	3513	681	80.3	72.1

Valence and arousal ratings were scaled between –1 and +1. Loudness levels and pitch values were computed using Matlab and Praat, respectively. (*Start and end periods were designated as the first and last second of the stimuli).

In the dot-probe task, where two stimuli are in direct competition, physical differences between the cues can influence the deployment of attention. Moreover, in a small sample of cues as in the current study (two stimuli for each affective category), physical factors might bias the results. In order to control for the physical differences between the cues, mean pitch and maximum loudness levels during the first and the last second of each sound were computed (Table 1), since extreme acoustical properties could influence attention. The pitch and the loudness of each sound in each cue pair and their differences were entered in a multiple correlation analysis to investigate possible interdependencies between them and the reaction times (RTs) measured during the experiment. The results yielded no significant correlations between the physical measures and the RTs. Thus, we argue that the changes in RTs are mostly due to the emotional categories rather than physical differences for the current data set.

### The Dot-probe Task

In the dot-probe task, two auditory stimuli (i.e., the auditory cues) were simultaneously presented at separate locations. Immediately after the sound offset either an auditory target was presented at the same location as one of the auditory cues, or there was no target (i.e., catch-trials; 33% of the trials). The target was a 100-ms long white noise burst, which was played back at 65 dBA. The task was to indicate the location of the target, as accurately and quickly as possible, by pressing a respective button on a keypad. Participants used the keypad with their both hands and pressed the buttons with their respective thumbs. Two different versions of the task were used depending on the sound source locations: left–right (LR) and front–back (FB) versions. In the LR version of the task, participants pressed the left button for locating a target on their left and right button on their right. In the FB version, the button locations were counterbalanced among participants (i.e., 12 participants pressed the left button for locating frontal targets, while the rest pressed the right button for frontal targets). Further, five participants were left-handed, which was taken into account during the counterbalancing of the button locations. We did not find any effect of button locations or handedness.

In the LR version of the task, the auditory cues and the target were presented through the loudspeakers located on the participants’ left and right hand side, while in the FB version loudspeakers were located directly in front of and behind the participants. All four loudspeakers (Genelec8020B) were located at 1.2-meter height from the ground (ca. at head-height when seated) and at 1.5-meter distance from the participants’ head. These locations were used to investigate whether any difference would occur in orienting toward lateralized targets compared to non-lateralized targets in the auditory domain.

### Procedure

**FIGURE 1:**
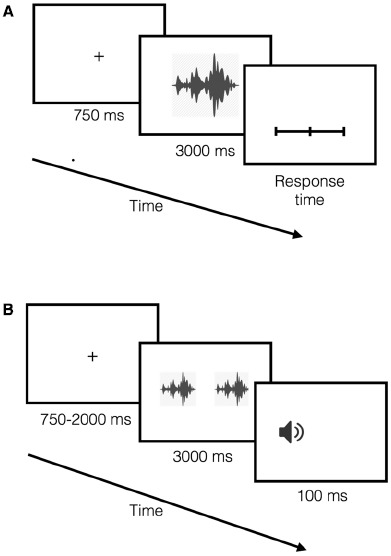
**The timeline of the experimental sessions for emotional reactions and the dot-probe task are plotted in (A) and (B), respectively. (A)** The auditory stimuli were presented after a fixation period. After each auditory stimuli participants reported their emotional reactions (indicated as response time in the plot). **(B)** The fixation period, whose duration was assigned randomly, preceded the simultaneous presentation of two task-irrelevant auditory cues. After the cues the target was presented; and the participants indicated the location of the target by pressing a button, as quickly and accurately as possible.

First, participants listened to the auditory stimuli, and were asked to rate how they felt when they heard each sound using separate visual analog scales (VASs) of valence (positive or negative content) and arousal (high or low arousal level). VASs were presented through an 8′′ LCD monitor placed directly below the front loudspeaker. Each trial started with a fixation cross (750 ms) that preceded the sound onset. Participants reported their emotional reactions following the sound offset. Next trial started 2 s after a response was registered (Figure 1A). Participants completed one experimental block consisting of each auditory stimulus presented once at each location (front, back, left, and right) in a random order.

After completing the first session participants started the dot-probe task. Each participant completed both versions of the task (i.e., FB and LR versions). The order of the task version was counterbalanced among the participants. In both versions of the task, each trial started with a fixation-cross whose duration was set randomly between 750 and 2000 ms; and it was followed by a simultaneous presentation of two task-irrelevant auditory cues. Participants were explicitly instructed to orient toward the fixation cross and not to move their heads during the trials. Maximum response time was 1500 ms following the target onset. Next trial started 3 s after either a response was registered or the response-window (1500 ms) was over (Figure 1B). For each trial, response and RT were recorded. In each version of the task, each one of the 36 possible pairs of sounds (including the same sound pairs) was presented three times, one of which was a catch-trial (no target). This resulted in 108 trials for each version of the task. For the target-trials (67 % of the total), the auditory target appeared at one of the two possible locations at equal times. Importantly, each auditory cue was followed by the target presented at the same location 50 % of the time. The emotional content of all pairs were fully randomized within each version of the task.

## Results

### Emotional Reactions

Valence and arousal ratings were scaled between –1 (negative or low arousal) and +1 (positive or high arousal) before they were submitted to separate three-factor repeated-measures analysis-of-variance (ANOVA) with sound source location (front, back, left, and right), affect category (negative, neutral, and positive), and stimulus (two in each category) as within-subject factors. Highly significant affect category main effects were found for both valence [*F*(2,44) = 141, *p* < 0.001, ${\text{η}}_{\text{p}}^{\text{2}}$ = 0.87] and arousal [*F*(2,44) = 51.2, *p* < 0.001, ${\text{η}}_{\text{p}}^{\text{2}}$ = 0.7]. Contrast analysis was employed to investigate the nature of the effect. For valence ratings only the linear contrast of the effect was significant [*F*(1,22) = 224, *p* < 0.001, ${\text{η}}_{\text{p}}^{\text{2}}$ = 0.91], which indicated that valence ratings were lowest for negative stimuli and highest for positive stimuli. Both linear [*F*(1,22) = 36.8, *p* < 0.001, ${\text{η}}_{\text{p}}^{\text{2}}$ = 0.63] and quadratic [*F*(1,22) = 57.9, *p* < 0.001, ${\text{η}}_{\text{p}}^{\text{2}}$ = 0.73] contrasts of the affect category main effect were significant for arousal ratings, which showed that induced-arousal was higher for emotional stimuli (negative and positive) compared to neutral stimuli, and that negative stimuli induced higher arousal than positive stimuli did (see Table 1 for means). The source location effect did not reach statistical significance.

Further, the results of the pair-wise comparisons between each stimulus indicated that valence and arousal ratings for emotionally neutral stimuli (i.e., yawning woman and microwave) did not differ significantly from each other. However, all the other stimuli had significantly different valence and arousal ratings from each other and from the neutral stimuli (at *p* < 0.01 level). Taken together, these results indicated that valence and arousal induction was successful.

### The Dot-probe Task

The localization accuracy of the target was near ceiling, which manifested itself in high hit rates (97 and 99% in FB and LR tasks, respectively) and zero false-alarms. Therefore, we only considered RTs (only-correct trials) in our analysis. The RTs were analyzed at three different levels: the trials that consisted of (1) auditory cues from different affect categories at different locations (48 trials in each version); (2) the same auditory cue at both locations (12 trials in each version); and (3) different auditory cues that belong to same valence category at both locations (12 trials in each version). In all the three levels, the two versions of the task were analyzed separately; and then, they were compared to each other.

#### Different Valence Cues

First, we focused on the trials that consisted of the auditory cues from different affect categories. Analysis provided no significant effects or interactions for positive-negative pairs. Hence, we focus on positive-neutral and negative-neutral pairs below (see Table 2 for average RTs).

**TABLE 2:** **Average reaction times for different valence pairs (95% CIs are indicated in parantheses)**.

**Target Location**	**Emotional stimulus location**	**Reaction times (ms)**	
		**Negative-neutral**	**Positive-neutral**
Front	Front	516 (68)	572 (74)
	Behind	589 (71)	578 (70)
Behind	Front	567 (61)	561 (77)
	Behind	540 (74)	541 (63)
Left	Left	429 (50)	467 (63)
	Right	447 (48)	466 (45)
Right	Left	448 (61)	430 (49)
	Right	425 (58)	466 (46)

Reaction times from the two versions of the task were analyzed separately using three-factor repeated-measures ANOVAs, with auditory cue pair (negative-neutral or positive-neutral), location of the emotional cue (i.e., location of the negative or the positive cue), and the target location as within-subject factors. For each cell in the ANOVA there were four different auditory cue-pairs in the experiment. Hence, the average RTs were used in the ANOVAs. In the FB version of the task, we found a significant interaction between the target location and the emotional cue location [*F*(1,22) = 10.57, *p* < 0.01, ${\text{η}}_{\text{p}}^{\text{2}}$ = 0.33], showing that RTs were faster when the target and the emotional cue were presented at the same location. Also, the three-way interaction was statistically significant [*F*(1,22) = 4.53, *p* < 0.05, ${\text{η}}_{\text{p}}^{\text{2}}$ = 0.17] indicating that the effect was larger for negative-neutral pairs (see Table 2 for mean RTs). The same three-way interaction was also found for the LR version of task [*F*(1,22) = 6.26, *p* < 0.05, ${\text{η}}_{\text{p}}^{\text{2}}$ = 0.22]. Moreover, the auditory cue pair had a significant main effect on the RTs during the LR task [*F*(1,22) = 6.42, *p* < 0.05, ${\text{η}}_{\text{p}}^{\text{2}}$ = 0.23], indicating that participants reacted faster on average for the negative-neutral pairs compared to the positive-neutral pairs.

**FIGURE 2:**
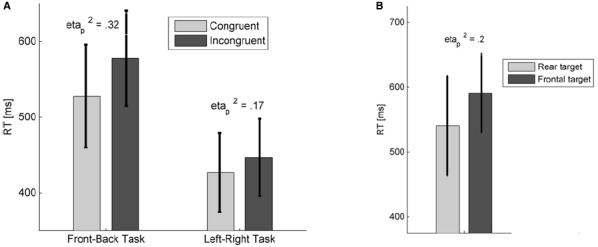
**(A)** Mean RTs from both versions of the dot-probe task, for negative-neutral auditory cue pairs. Congruent trials are those when the negative auditory cue and the target were presented at the same location. Effect sizes and 95% confidence intervals were also shown. **(B)** Mean RTs for the same stimulus cue-pair trials in the front-back version of the task. Error bars indicate 95% confidence intervals.

When the analyses were made after cue pairs were separated, we found a significant interaction effect between the target location and the emotional cue location only for negative-neutral pairs in both FB [*F*(1,22) = 10.41, *p* < 0.01, ${\text{η}}_{\text{p}}^{\text{2}}$ = 0.32] and LR [*F*(1,22) = 4.65, *p* < 0.05, ${\text{η}}_{\text{p}}^{\text{2}}$ = 0.17] versions of the task (Figure 2A). The effect did not reach significance for positive-neutral pairs in either task versions. We also found that the reaction-times were significantly shorter for the LR version of the task compared to the FB version [*F*(1,22) = 21.8, *p* < 0.001, ${\text{η}}_{\text{p}}^{\text{2}}$ = 0.5].

#### Same Stimulus Cues

For the analysis of the trials where the same auditory cue was presented in both locations, we first considered the differences between the auditory stimuli. The RTs were submitted into ANOVAs with target location and stimulus (six auditory cues) as within-subject factors. The analysis did not provide any significant main effects or interactions for either version of the task. Moreover, pair-wise comparisons did not yield any significant difference in RTs between the auditory cues. Then, the average RTs from the three affect categories (two stimuli in each category) were submitted into separate ANOVAs with the target location and the affect category (positive, neutral, and negative) as within subject factors. There were no significant main effects or interactions in the LR version of the task. In the FB version, however, the target location [*F*(1,22) = 5.6, *p* < 0.05, ${\text{η}}_{\text{p}}^{\text{2}}$ = 0.2] had a significant main effect on the RTs, indicating that the participants were faster in localizing the target when it appeared behind them compared to in front of them (Figure 2B). We have also carried out an ANOVA with affect category (negative, neutral, and positive), sound source location (front and back), and stimuli (two in each category) factors to study whether there was a difference in affective reactions between stimuli that were located in the frontal and the rear auditory space. The analysis did not provide significant main effect of sound source location on either arousal or valence ratings. However, a significant linear contrast of the interaction between the affect category and the source location on self-reported arousal indicated that the more negative the stimuli the higher the arousal they induced when they were presented behind the listeners [*F*(1,22) = 5.42, *p* < 0.05, ${\text{η}}_{\text{p}}^{\text{2}}$ = 0.2]. Finally, after comparing the two versions of the task, it was found that participants were significantly faster in LR version compared to FB version [*F*(1,22) = 14.12, *p* < 0.01, ${\text{η}}_{\text{p}}^{\text{2}}$ = 0.39].

#### Same Valence Cues

Reaction times were analyzed for the auditory cue pairs from the same affect category using two-factor repeated-measures ANOVAs with the affect category (positive, neutral, and negative) and the target location as within-subject factors. We could not find significant main effects or interactions in either version of the task. Overall, the participants were faster in the LR version compared to the FB version [*F*(1,21) = 16.04, *p* < 0.01, ${\text{η}}_{\text{p}}^{\text{2}}$ = 0.43].

## Discussion

The present study set out to investigate whether the emotional salience of auditory stimuli could be used as an exogenous cue for spatial orienting of attention. We adapted the dot-probe task to the auditory modality (Posner, 1980). The results showed that task-irrelevant negative sounds when paired with neutral sounds provided exogenous cues for the spatial attention. However, the same effect could not be found for positive stimuli that were paired with neutral sounds; and there were no indication of exogenous orientation of attention for positive/negative cue pairs. This could be due to the varying influences positive and negative emotional stimuli have on attention. It has been suggested that negative affect acts to focus the attention on specific stimuli, while positive affect broadens the attention (Pourtois et al., 2004; Rowe et al., 2007). Further, same valence cues (negative, positive, or neutral) did not affect the task performance. We argue that this is due to the smaller difference in emotional arousal between the same valence events. For the current stimulus set, the largest differences in elicited emotional arousal are between negative and neutral sounds, where we could observe an attentional orienting effect. As the arousal difference diminishes between the competing auditory cues the orienting effect seems to disappear. In other words, emotional arousal induced by an event may lead to the attentional prioritization (Vuilleumier, 2005; Mather and Sutherland, 2011). Taken together, our study provides clear behavioral evidence that task-irrelevant negative emotional stimuli provide cues for orientation of auditory spatial attention. Even though, similar results were shown in visual domain, they are scant in auditory modality. Further, adapting the dot-probe task to auditory modality we could explore different portions of the space surrounding the individual that is more ecologically valid compared to the classical version of the task in the visual modality (e.g. left or right side of a fixation point).

Spatial cueing studies using dot-probe task has been mostly used in visual modality for both healthy and clinical groups. Early research provided evidence for the spatial attention bias in favor of negative (mostly threatening) over neutral stimuli in both anxious patients and high-trait anxious individuals (for a review, see Yiend, 2010). Anxiety seems to be associated with a preferential bias for negativity. Over the years the bias for negativity were found in different clinical groups such as depression (Laeger et al., 2012) and job burnout (Sokka et al., 2014). The successful application of the dot-probe task in the auditory modality may also provide a further methodological tool for research in clinical populations.

We also found that the participants were significantly faster during the LR version of the task compared to the FB version. Psychophysical evidence suggests that humans use intensity and arrival time differences at respective ears to locate sounds, i.e., binaural cues (Blauert, 1997). For locations in the median plane those differences do not occur. Hence, localization cues are only provided by the spectral modulations caused by the head, torso and the shape of outer ear. However, those monaural cues are highly frequency-dependent. Due to the lack of binaural cues, participants might have needed longer time to locate the target in the FB version of the task in general.

Further, in the FB version of the task participants were faster in locating the target occurring behind when the same stimulus was played in both locations. The results also showed that the more negative the stimuli were, the higher the arousal they induced when they were presented behind the listeners. These results may point toward an attentional bias for the rear auditory space. In a recent study, we found evidence for a possible auditory bias toward the rear perceptual field at both attentional and emotional levels (Asutay and Västfjäll, 2015). The results of a rapid localization experiment showed that both localization speed and accuracy were higher, and stronger negative emotional reactions were induced when the sounds occurred behind participants compared to when they occurred in front of them. Further, previous research reported that sounds occurring behind the participants tended to induce higher arousal (Tajadura-Jiménez et al., 2010), and that emotionally neutral changes in a complex auditory-scene could be detected more accurately when they occurred behind the listeners compared to in front of them (Asutay and Västfjäll, 2014). It has been suggested that the auditory system has an alarm function (Juslin and Västfjäll, 2008), and that the spatial processing in the auditory-dorsal pathway has a function of guiding the visual system to a particular location of interest (Arrnott and Alain, 2011). Hence, an attentional bias for the rear auditory field can be useful in the case of emotionally significant information. In fact, the cross-modal spatial attention studies indicated that the audiotactile interactions occurring in rear space tend to trigger defensive head and arm movements, and are qualitatively different from those occurring in the frontal space (for a review, see Occelli et al., 2011). This indicates that frontal and rear space representations may trigger differential sensory response properties (Occelli et al., 2011). The present study lends further evidence for this effect, although it was not found for conditions where different stimuli were presented at different locations. Thus, it seems that the attentional bias toward the rear auditory field, which has a small effect size, was not observable unless the temporal and spectral variation of the stimuli was the same at both locations. Testing of this particular effect could shed further light on this potential bias.

In sum, the results of the current study indicated that the auditory spatial attention is influenced by emotional salience of the stimuli (mainly negative emotion) and their locations relative to the observer’s body. We argue that one of the primal functions of the auditory system is to detect salient changes and alert the organism to shift attention if necessary; and that affect is an integral part of auditory attention and perception. The findings are also consistent with the research suggesting that emotional information provides invaluable cues for allocation of attention and mental resources (Vuilleumier, 2005; LeDoux, 2012).

## Author Contributions

Both authors contributed to the development of the study concept and design. Data collection and analyses were performed by EA drafted the manuscript and DV provided critical revisions.

### Conflict of Interest Statement

The authors declare that the research was conducted in the absence of any commercial or financial relationships that could be construed as a potential conflict of interest.
